# Chronic obstructive pulmonary disease and heart rate variability: a literature update

**DOI:** 10.1186/1755-7682-7-43

**Published:** 2014-10-03

**Authors:** Adriano L Roque, Vitor E Valenti, Thais Massetti, Talita Dias da Silva, Carlos Bandeira de Mello Monteiro, Fernando R Oliveira, Álvaro Dantas de Almeida Junior, Sheylla Nadjane Batista Lacerda, Gustavo Carreiro Pinasco, Viviane Gabriela Nascimento, Luiz Gonzaga Granja Filho, Luiz Carlos de Abreu, David M Garner, Celso Ferreira

**Affiliations:** Post-graduate Program in Cardiology, Federal University of São Paulo – Paulista School of Medicine, 715, Pedro de Toledo St, Vila Clementino, São Paulo, SP 04039-032 Brazil; Post-graduate Program in Physical Therapy - Faculty of Science and Technology UNESP, Presidente Prudente, SP Brazil; Post-graduate Program in Rehabilitation Sciences - Faculty of Medicine, University of São Paulo, São Paulo, SP Brazil; Department of Public Health, Laboratory Design of Studies and Scientific Writing, School of Medicine of ABC, FMABC, Santo André, SP Brazil; Department of Environmental Health, Harvard School of Public Health, Boston, MA USA; Cardiorespiratory Research Group, Department of Biological and Medical Sciences, Faculty of Health and Life Sciences, Oxford Brookes University, Gipsy Lane, Oxford OX3 0BP UK

**Keywords:** Autonomic nervous system, COPD, Heart rate variability, Cardiovascular system

## Abstract

**Background:**

The literature indicates that chronic obstructive pulmonary disease (COPD) affects cardiac autonomic control. In this study, we conducted a literature review in order to investigate the heart rate variability (HRV) in COPD subjects.

**Methods:**

A search was performed in Medline database, using the link between the keywords: “autonomic nervous system”, “cardiovascular system”, “COPD” and “heart rate variability”.

**Results:**

The search resulted in a total of 40 references. Amongst these references, the first exclusion resulted in the barring of 29 titles and abstracts, which were not clearly related to the purpose of review. This resulted in a total of 11 articles that were then read and utilized in the review. The selected studies indicated that there is significant reduction of HRV in patients with COPD, characterized by reduction of indices that assess parasympathetic activity in addition to dealing with the global autonomic modulation. We also established that supervised exercise can reduce these harmful effects in COPD patients. Also, it was reported that the use of non-invasive ventilation in these patients may contribute to the improvement of respiratory symptoms, with no impairing, and may even induce positive responses in cardiac autonomic regulation.

**Conclusion:**

The studies indicate a need for further investigations to guide future therapies to improve the treatment of cardiovascular system in the respiratory diseases.

## Introduction

Chronic obstructive pulmonary disease (COPD) is defined as a respiratory illness that can be prevented and treated. COPD is characterized by chronic airflow obstruction which is not fully reversible [1, 2] and significant systemic manifestations such as: nutritional depletion, structural and functional alterations of the respiratory and peripheral skeletal muscles and arrhythmia [3].

There are studies addressing changes in sympathetic-vagal balance caused by cardiopulmonary diseases, such as COPD. These could be identified by the presence of harmful alterations in the autonomic nervous system (ANS) function of subjects with COPD [4, 5]. Because it is a disease with high morbidity and mortality, and coexists with cardiovascular disease, it is necessary a higher level of care in these patients.

The heart rate variability (HRV) has been widely used to assess the behavior of ANS, it is a noninvasive, inexpensive, and can describe a phenomena related to ANS in healthy and unhealthy subjects [6]. It is a conventionally accepted term to describe the fluctuations in the intervals between consecutive heartbeats (RR intervals), and is directly related to the performance of the ANS on the sinusal node [7]. It implies that an individual with low HRV has greater pre-disposition to submit cardiovascular problems, under physiological conditions, the greater HRV, better the health condition of the subject [8].

HRV analysis has been studied since 1965 and has been increasingly emphasized for being an important method for early diagnosis of cardiovascular diseases [6]. Previous studies have indicated reduced HRV in patients with COPD compared to healthy individuals of the same age [4, 9], showing inferior clinical prognosis of these individuals. Based on these concepts, the present study aimed to investigate the relationship between COPD and HRV.

The purpose of this study was to investigate the established results about “autonomic nervous system”, “cardiovascular system” and “COPD”, and “Heart Rate Variability” and “COPD” in previous studies.

## Methods

### Search strategy

**Figure 1:**
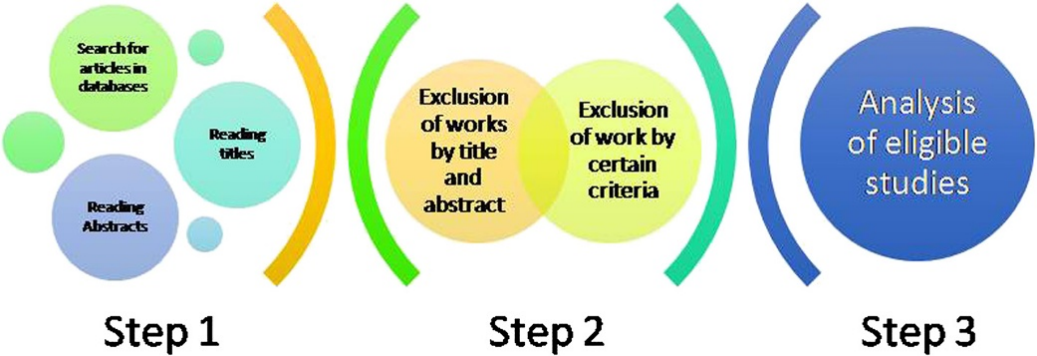
**Step by step showing the literature review.**

**Figure 2:**
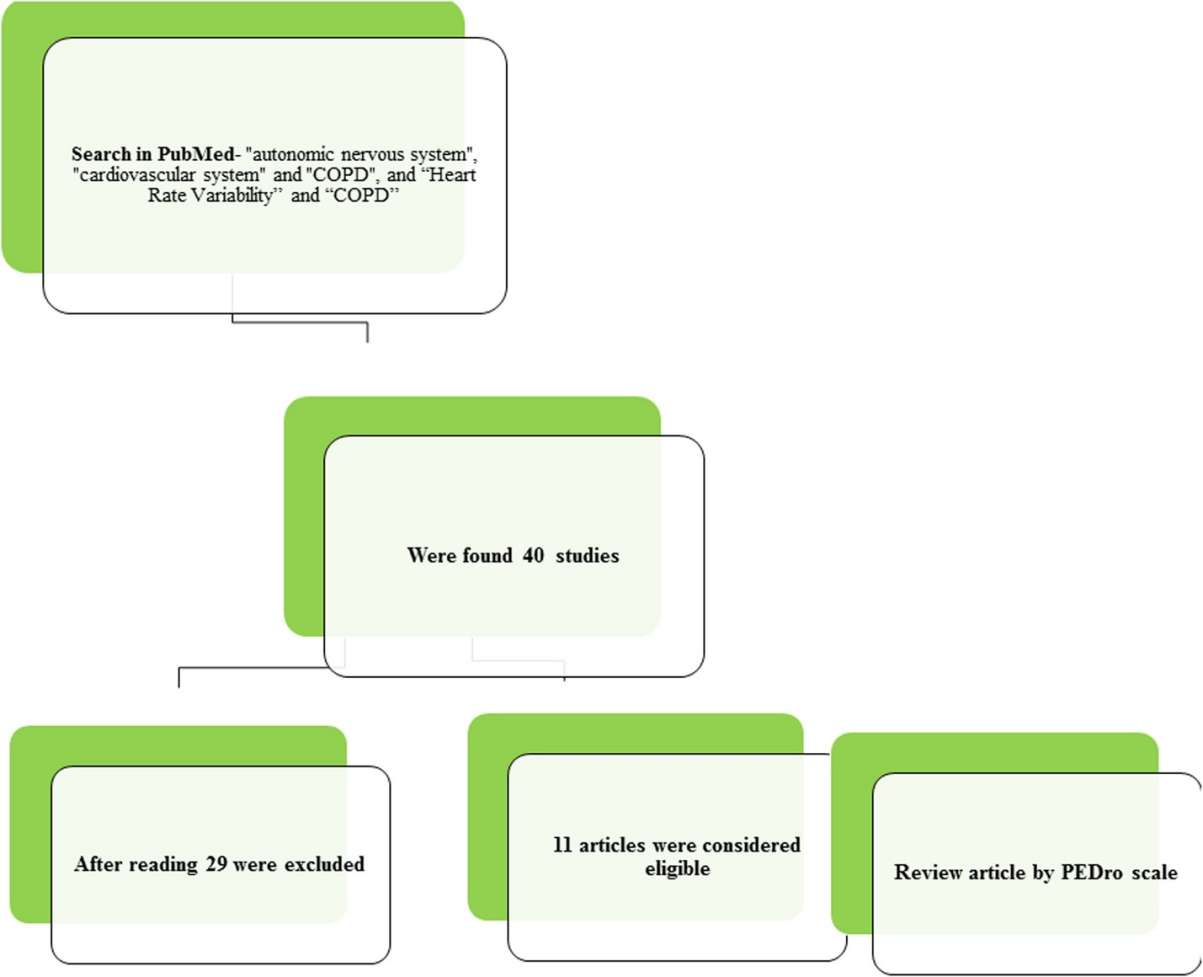
**Flowchart of search strategy and selection process.**

All steps are presented in Figures 1 and 2. The review was analyzed between March 2014 and May 2014. Data from Medline (via PubMed), databases were searched using the following keywords: “autonomic nervous system”, “cardiovascular system” and “COPD”, and “Heart Rate Variability” and “COPD”. These were defined by the Medical Subject Headings (MeSH) in the National Library of Medicine.

### Inclusion and exclusion criteria

We excluded studies that did not show full text or abstract in English between 2009 and 2014 and review of literature as well as summaries of academic Dissertations or Theses. Inclusion criteria were considered clinical and basic studies that investigated the behavior of the autonomic nervous system in patients with chronic obstructive pulmonary disease (COPD), published in the last 6 years.

### Selection strategy

Initially a screening of titles was undertaken related to the topic. This selection was based on evidence that related cardiac autonomic control (via heart rate variability) with chronic obstructive pulmonary disease (COPD). At the end of the study, we excluded duplications, as this was done in several databases. Reading the detailed summaries of articles in order to select those that addressed only the behavior of the autonomic nervous system via some protocol in patients with COPD was undertaken. We excluded abstracts not related to the issue, the full texts were evaluated and those who did not fit the exclusion criteria were included as the final result of the research.

To increase confidence in the selection of articles, all potentially relevant articles were reviewed independently by two researchers, who after reading through all of them, entered consensus to establish which articles fulfilled the inclusion criteria [10].

### Data analysis

Data was qualitatively evaluated and presented in tables with a description of the following profile: author and year of study and the main conclusions found by the authors during the studies.

There are many scales that help the studies evaluation, nowadays; the most used in rehabilitation area is the PEDro [11] scale. This scale was developing by Physiotherapy Evidence Database to be used in experimental studies and has a total score of 10 points, including evaluation criteria of internal validity and presentation of statics analysis used [11].

In order to demonstrate the methodological quality of the studies it was considered the article with good level of evidence the one with score equal or higher than 6 according with evaluation scale PEDro. This criterion was based on Snider’s work [12], that consider studies scoring 9-10 on the PEDro scale are methodologically ‘Excellent’, 6-8 ‘Good’, 4-5 ‘Fair’ and below 4 ‘Poor’.

## Results

The electronic search resulted in a total of 40 references. Among these references, the first elimination resulted in the exclusion of 29 titles and abstracts, which were not clearly related to the purpose of review. It resulted in a total of 11 articles that were then fully read and utilized in the review.

Initially, there were 40 studies found, of these 29 articles were excluded to not fit the inclusion criteria (Figure 1). The data extracted from the 11 eligible (Table 1).

**Table 1:** **Main studies about the influences of chronic obstructive pulmonary disease on cardiac autonomic control**

Selected articles	Principal outcomes	Score Pedro
**Van gestel et al., 2011** [13]	We studied 60 patients with COPD and measured HRQL, as assessed by the Chronic Respiratory Disease Questionnaire, and cardiac autonomic dysfunction, as assessed by heart rate variability (HRV). Analysis of HRV was performed using a Holter-ECG device during a recording period of 5 min. To evaluate a possible association between these factors, univariate and multivariate analyses were used. Resting parasympathetic tone, as measured by HRV, is independently associated with HRQL, which emphasizes the role of cardiac autonomic dysfunction on HRQL in patients with COPD.	3/10
**Gunduz et al., 2009** [14]	Twenty five moderate to severe COPD patients and 25 healthy subjects were included in this study. Pulmonary function tests and echocardiographic examination, arterial blood gases analysis were performed; HRV and HRT analysis were assessed from a 24-hour Holter recording. In addition to HRV parameters, HRT onset was significantly different in COPD patients. In our opinion, the combination of HRV variables and HRT onset may be simple and elegant ways of evaluating cardiac autonomic functions.	3/10
**Cheng et al., 2014** [15]	Sixty-four patients with COPD participated in a 12-week, 2 sessions-per-week, hospital-based PR program. Baseline and post-PR status were evaluated by spirometry, HRV, health-related quality of life, cardiopulmonary exercise test, respiratory muscle strength, and dyspnea Borg's scale. PR results in significant improvements in autonomic function, with concurrent improvements in HRQL and exercise capacity.	4/10
**Suh et al., 2013** [16]	The study utilized a 2 (disease status) × 2 (anxiety group) factorial design examining HRV associated with anxiety symptoms and COPD during a standardized acute social stress task. 30 COPD patients were age- and gender-matched with 30 healthy controls. Anxiety is associated with dysregulated HRV response to a psychosocial stressor, but the negative influence of anxiety and COPD on autonomic function did not appear to be additive. Comorbid anxiety in patients with COPD is associated with increased behavioral and psychological symptoms of distress.	5/10
**Dias de Carvalho et al., 2011** [17]	We evaluated geometric index of HRV in COPD subjects. We analyzed data from 34 volunteers, divided into two groups according to spirometric values: COPD and control. For analysis of HRV indexes the volunteers remained in the supine position for 30 minutes. Subjects with COPD present reduction of geometric indexes of HRV, indicating reduced heart rate variability.	6/10
**Mendes et al., 2011** [18]	To analyze the behavior of heart rate (HR), blood pressure (BP) and heart rate variability (HRV) during the FVC test in COPD patients. Nineteen men with COPD performed the FVC test while having their HR monitored. HRV was assessed in time and frequency domains at rest, before and after the best FVC maneuver. BP was measured at rest, immediately before and at the end of the test, as well as 10 minutes after the end of the test. The FVC test influences the behavior of COPD patient HR without changing autonomic control or BP.	4/10
**Camillo ca et al., 2011** [19]	We aimed to investigate changes in HRV after two exercise training programs in patients with COPD. Forty patients with COPD were randomized into high (n = 20) or low (n = 20) intensity exercise training (3-month duration), and had their HRV assessed by the head-up tilt test before and after either protocols. High-intensity exercise training improves HRV at rest and during orthostatic stimulus in patients with COPD.	5/10
**Carvalho et al., 2011** [20]	We analyzed data from 30 volunteers, who were divided into two groups according to spirometric values: COPD (n = 15) and control (n = 15). For analysis of HRV indices, HRV was recorded beat by beat with the volunteers in the supine position for 30 minutes. COPD subjects present reduced short-term fractal correlation properties of HRV, which indicates that this index can be used for risk stratification, assessment of systemic disease manifestations, and therapeutic procedures to monitor those patients.	5/10
**Reis et al., 2010** [21]	Ten COPD patients and nine age-matched healthy volunteers participated in this study. Heart-rate variability (HRV) was obtained at rest and during respiratory sinusal arrhythmia maneuver (RSA-M) by electrocardiograph. Patients with chronic obstructive pulmonary disease presented impaired sympathetic-vagal balance at rest. In addition, cardiac autonomic control of heart rate was associated with inspiratory muscle weakness in chronic obstructive pulmonary disease.	3/10
**Borghi-Silva et al., 2009** [22]	Forty patients of both sexes with moderate-to-severe COPD were randomly allocated to aerobic exercise training (PT, n = 20) or to usual care (Control, n = 20). The training program consisted of lower and upper limb stretching and 30 min of treadmill exercise, 3 times per week for a 6-week period. The improvement in submaximal performance after exercise training was associated with parasympathetic activity.	4/10
**Antonelli Incalzi et al., 2009** [23]	We studied 54 patients with COPD. Heart rate variability (HRV) was assessed based on 24-h Holter ECG recording. Sympathetic modulation decreased for increasing severity of COPD. In conclusion, drawing impairment correlates with depressed sympathetic modulation in patients with COPD, and both might be indexes of COPD severity.	3/10

## Discussion

During the study of the articles used in the preparation of this review, it was observed that patients with COPD have reduced heart rate variability compared to healthy patients. Moreover, some features, such as the non-invasive ventilation can improve the cardiac autonomic control of these individuals suggesting a reduction in the impact of cardiac co-morbidities therein. In addition, we verified that some studies have analyzed the effects of exercises like strength and endurance exercises; six minute walk test or even through pulmonary rehabilitation, and it was observed that all interventions improve heart rate variability in COPD patients, which cautions us to importance of physical exercise to improve the quality of life of these population.

Heart rate variability has been used as a tool to analyze the behavior of the autonomic nervous system on the heart, and compare possible differences between healthy and unhealthy people. Its decrease is related to increase morbidity and mortality [24], which was confirmed the data found in our research on COPD. Studies indicate that there are changes in HRV in a list of cardiorespiratory disorders. COPD is associated with vascular remodeling that modifies the pulmonary circulation. This pathological mechanism is usually caused by hypoxia generated by the disease [25].

According to the study of REIS et al [21], both patients with COPD and patients with congestive heart failure (CHF), exhibit alterations in autonomic modulation of heart rate at rest and during respiratory sinusal arrhythmia maneuver (RSA-M) compared with apparently healthy individuals. During rest, patients with COPD showed a reduction in sympathetic activity compared to the control group. Regarding the CHF it was also observed decreased sympathetic and parasympathetic activity of the subjects. It was indicated a possible relationship to regulatory changes of autonomic centers, the sensitivity of chemoreceptors or respiratory pattern characterized by periodic oscillations presented by these patients. During the RSA-M it was observed that both COPD patients and patients with CHF, through the analysis of HRV indices in the time domain (SDNN and rMSSD) and frequency domain (LF and HF, both in units absolute) had reduced response, revealing a decrease in HRV compared with control subjects. These data presented in the study indicated that it is possible that reduced HRV observed in patients of both groups may be related to changes in lung compliance and response of lung receptors stretches.

A study of Mendes et al [18], evaluated the heart rate, blood pressure and heart rate variability in test forced vital capacity (FVC) in patients with COPD and demonstrated that significant changes in HR, while on BP and HRV it did not occurred the same changes. Throughout the test, HR was undergoing changes as fall during the beginning of the inspiratory phase of the test, followed by an increase until the end of it, with the end of the inspiratory phase HR was reduced below the HR at rest before resuming their baseline. During the expiratory phase, HR was progressively increasing, the authors suggested this to be related to the increased intrathoracic pressure and decreased venous return, and reduction in vagal activity and increased sympathetic activity that occurs during expiration. When assessing the HRV, the absence of significant changes was verified, and the authors highlighted the short duration of the maneuver, the age of the volunteers and a probable autonomic dysfunction of individuals.

By analyzing the geometrical HRV indices, Dias de Carvalho et al [17] illustrated decreased SD1, SD2, TINN and RRtri in the COPD group, while the SD1/SD2 ratio were similar between the two groups. These findings suggest a reduced HRV in patients with COPD. Reducing the SD1 index indicates reduction in vagal activity of these individuals, since the collapse in values of SD2, TINN and RRtri in patients with the disease compared with healthy subjects, indicating overall reduction in autonomic modulation of these individuals, which supports other studies which indicated global autonomic damage in COPD. Through the analysis of the Poincaré plot, the authors found greater dispersion of data related to patients with COPD, which, again, suggests the same reduced in HRV. It was highlighted the importance of supervised exercise in subjects with COPD, since the literature shows these beneficial effects on cardiac autonomic modulation.

In a study by Dias de Carvalho et al [20], it was analyzed the properties of fractal correlation in patients with COPD, with loss or breakdown of short-term fractal correlation properties of HRV (alpha-1) associated with the reduction of sympathetic and parasympathetic activity in patients with COPD. According to the authors, patients with COPD exhibit dynamic changes in HR indicating loss of chaotic response. Equally, linear indexes in time and frequency domain analyzed in the study illustrated a reduction in sympathetic and parasympathetic activity in volunteers of the COPD group compared to the control group. The authors have attributed to this loss several clinical events such as heart failure and acute myocardial infarction. It was also suggested that individuals with COPD exhibit autonomic dysfunction, indicated by the loss of short-term fractal correlation properties and reductions in global activity of HRV, supporting the importance of using these tools for evaluation of morbid states, because the ability to evaluate the loss of homeostasis of patients, allowing better discrimination between individuals with or without physiological change. With respect to long-term exponents (alpha-2) and alpha-1/alpha-2 ratio, there was no difference between the COPD group and the control group.

In order to analyze HRV after two exercise programs in patients with COPD, Camillo and coworkers [19]assessed 40 patients divided into two groups: high intensity, characterized by endurance exercise and strength, and of low intensity, that consisted of calisthenics and breathing exercises for a period of 3 months. Cardiac autonomic control was assessed before and after 3 months of training and, was judged during rest and as a response to an orthostatic stimulus using a head-up tilt test following a previous published protocol. Patients were positioned on an orthostatic table where they initially stayed for 10 minutes in supine position. The table was then lifted 75°, and this was followed by a period of more 10 minutes in orthostatic position. The authors concluded that three months of high-intensity exercise training enable an important improvement in post-training cardiac autonomic function in patients with COPD. Additionally, better baseline values of upper limbs muscle force, physical activity in daily life and total heart rate variability may help predicting those patients who will more likely improve their cardiac autonomic function after a high-intensity exercise training program.

An important study [15] attempted to investigate the effect of pulmonary rehabilitation on heart rate variability during exercise in patients with COPD. Beyond, the effect of pulmonary rehabilitation on health-related quality of life and exercise capacity concurrently. Cardiac autonomic control was measured with a total recording time of 5 minutes at rest and at peak exercise and, were obtained from the ECG signals. The protocol program consisted of 12-week outpatient-based program with two sessions per week. Before all training sessions, patients went through prior educational training, then the lower limb cycle ergometer exercise was performed. The training protocol of the lower limb exercise consisted of warm up of 4 minute, followed by 60-100% peak VO_2_ for 40 minute and finally cool down of 4 minute. Work rate, SpO_2_, HR, BP, dyspnea Borg’s scale, and leg fatigue during the exercise training were monitored. They concluded that patients with COPD often have impaired parasympathetic and sympathetic activity and poor exercise capacity, and quality of life. Pulmonary rehabilitation provides significant improvement in HRV with concurrent improvements in HRQL and exercise capacity.

In an attempt to investigate heart rate variability responses to a psychosocial stressor in COPD patients, and the potential role of anxiety as a confounding factor in this relationship, Suh and colleagues [16] analyzed four groups of participants: COPD patients with elevated anxiety, COPD patients without anxiety, healthy individuals with elevated anxiety, and healthy individuals without elevated anxiety with 15 participants each. Participants performed a test to check the level of anxiety and eligible completed the pulmonary function test. The study was divided into three phases: Baseline, Task, and Recovery. At Baseline, all participants completed a packet of self-report questionnaires. Following the questionnaires, participants remained sitting quietly for 5 minutes to obtain a stable measure of heart rate variability at rest. During the Task phase, participants were exposed to the stressor task. A modified version of the Trier Social Stress Test was used for this study. And finally during the Recovery phase, each participant completed a post-stressor questionnaire of state anxiety. The participant then listened to relaxing music for 20 minutes. In this study the authors indicate that the combined effect of anxiety and COPD did not have a cumulative negative effect on autonomic function, contrary to the original hypothesis. However, an atypical pattern of HRV in response to the stressor task for the COPD patients with elevated anxiety and healthy individuals with elevated anxiety compared to their non-anxious counterparts suggests that anxiety may play a mediating role in HRV patterns in response to a stressor.

Hypothesizing that respiratory muscle weakness negatively influences heart rate variability during respiratory maneuvers in patients with COPD; Reis and coworkers [26] aimed to evaluate the influence of respiratory muscle strength on the magnitude of respiratory sinus arrhythmia. All chronic obstructive pulmonary disease patients used short-action bronchodilators, and six used long-action bronchodilators. Subjects in the control group were free of chronic pulmonary, cardiovascular, immune, and/or metabolic disease. The volunteers were kept at rest in the supine position for approximately 10 minutes to ensure that a true resting HR value was achieved. Then, the ECG signal and the instantaneous HR were obtained at rest in the supine position for 15 minutes. Subsequently, the heart rate and RR intervals were recorded during the respiratory sinus arrhythmia maneuver in the supine position in the following order: for one minute at rest with spontaneous breathing; for four minutes while performing the respiratory sinus arrhythmia maneuver; and for one minute at rest with spontaneous breathing. It was concluded that COPD patients showed evidence of impaired autonomic modulation of heart rate at rest and during respiratory sinus arrhythmia maneuver, the relationship between the maximal inspiratory pressure and heart rate variability indices during respiratory sinus arrhythmia maneuver indicates that the inspiratory muscle weakness observed in this population may be associated with cardiac autonomic control.

The aim of the study by Borghi-Silva and coworkers [22] was to assess the impact of an aerobic exercise program on autonomic modulation in patients with moderate-to-severe COPD. Forty patients of both sexes were included in the study and all of them received regular treatment consisting of inhaled bronchodilators and steroids and none of them were prescribed oral steroids, antibiotics, antihypertensive or beta-blockers. Heart rate variability data were collected during 10 minutes of rest and throughout the six-minute walk test. Aiming to maintain peripheral oxygen saturation greater than 90%, four patients of the experimental group and three patients in control group received supplemental oxygen. Concluding, the study demonstrates that a 6-week aerobic exercise training program leads to an improvement in exercise tolerance, promotes ventilator and physiological adaptations and favorably impacts the derangements in autonomic modulation of heart rate both at rest and during exercise in patients with COPD.

A group of Italian researchers [23] studied 54 patients with hypoxemic chronic obstructive pulmonary disease. Neuropsychological assessment was performed by The Mini Mental State Examination and the Mental Deterioration Battery and heart rate variability was assessed based on 24-hour Holter ECG recording. It was observed by the researchers, a correlation between drawing impairment and depressed sympathetic modulation of the neuroautonomic tone. Such a relationship seems worthy of reassessment in a larger and more heterogeneous COPD population in the framework of properly designed studies including also a well balanced set of executive and copying tests. Confirming or denying present findings would make physicians aware of whether a further dimension should be added to the many characterizing COPD as a systemic disease.

An important study of Van Gestel and co-workers [13] aimed to evaluate whether there is an association between cardiac autonomic dysfunction and health-related quality of life in COPD patients. They applied a six-minute walk test in all patients and performed on a 30 metre indoor track using standardized encouragement strategy. Breathlessness and leg fatigue were assessed using a conventional Borg scale in German. Analysis of heart rate variability was performed using a Holter-ECG device for a recording period of 5 minutes, with the patients at rest, sitting on a chair. Cardiac autonomic function was performed 30 minutes after pulmonary function was assessed during the midday between 11:00 –15:00. The study shows that resting parasympathetic tone, as measured by HRV, is independently associated with health-related quality of life emphasizing the role of cardiac autonomic dysfunction on health-related quality of life in patients with COPD. Modification of cardiac autonomic dysfunction may therefore be of benefit in the treatment of COPD patients, but this needs to be proven in controlled interventional trials.

Gunduz and colleagues [14] also analyzed the presence of autonomic dysfunction in patients with COPD by heart rate variability and heart rate turbulence analysis and to determine whether the parameters of these in this population are different from the normal population. Spirometric and blood tests were performed in all participants and heart rate variability was assessed by 24-hour Holter monitoring. In the view of authors, the combination of heart rate variability and heart rate turbulence variables may be simple and elegant ways of evaluating cardiac autonomic functions. Such a combination may increase the positive predictivity and lead to a more accurate identification of high risk patients, more aggressive treatment towards preventing sudden death and/or preventing progression of disease to mortality.

By analyzing the studies eligible for our review, unfortunately, we found poor methodological quality of most of them, according to the PEDro scale [11]. We observed that 36% of them had score 3, 27.5% score 4, 27.5% score 5, and only one study with score 6, considered with good methodological quality; it should be emphasized that better designed studies can better represent proposed objectives by their results.

Thus, HRV is an important tool for assessing the autonomic nervous system (ANS), which has an important role in maintaining homeostasis. Its use is diverse and it stands as a predictor of the internal functions of the body, both in normal and pathological conditions, characterizing current instrument evaluation and identification of problems in the health, growth and human development [27]. Health care is conventionally regarded as the diagnosis, treatment, and prevention of disease, illness, injury, and other physical and mental impairments in humans. How we define the quality of public health at any given time must be compatible with future generations enjoying health in an equivalent way [28].

## Conclusion

We present in this review studies that focused on the behavior of the HRV in patients with COPD during interventions, such as, respiratory sinusal arrhythmia maneuver, non-invasive ventilation, among others. These data show the importance of HRV as low cost method for early diagnosis of cardiovascular diseases concomitant with lung diseases, since these diseases tend to coexist influencing on morbidity and mortality of these patients. It is well known that COPD patients tend to have a reduced HRV, and consequently deterioration of symptoms. These studies attempt to show the importance of interventions that help to minimize the effects that COPD may lead in these patients.
